# Neoplasm of a supernumerary undescended testis: A case report and review of the literature

**DOI:** 10.1016/j.ijscr.2018.10.082

**Published:** 2018-11-13

**Authors:** Hamza Boussaffa, Sahbi Naouar, Nidhal Ati, Mohamed Amri, Badereddine Ben Khelifa, Braiek Salem, Rafik El Kamel

**Affiliations:** Urology Department, Ibn El Jazzar Teaching Hospital, Kairouan, Tunisia

**Keywords:** Supernumerary testis, Undescended testis, Neoplasm, Case report

## Abstract

**Introduction:**

Polyorchidism, defined as the presence of more than two testicles, is a rare congenital abnormality of the male genital tract. There is no consensus regarding the management of supranumerary testis (SNT) due to its rareness. To the best of our knowledge, this is the first report of leiomyoma in SNT.

**Presentation of case:**

We report the case of a 41-year-old man with two right testes sharing a common vas deferens and separate epididymides. Orchiectomy of right testes was performed. Pathology examination found that the superior testis was the site of a leiomyoma and the lower tesits was the site of an intratubular germ cell neoplasia (IGCN). Orchiopexy and biopsy were later performed for the left testis. Histology was compatible with IGCN. We opted for follow-up to avoid risks of hypogonadism.

**Discussion:**

Polyorchidism is usually asymptomatic and found incidentally during surgery for inguinal hernia, undescended testes as in our case, torsion, hydrocele or testicular tumor. If the SNT is scrotal, and there is no other indication for surgery, most authors recommend conservative management with regular ultrasound follow-up. If nonscrotal SNT is found incidentally during surgery, orchiectomy could be performed because of increased risk of malignancy. Treatment of IGCN includes surveillance, orchiectomy, or low-dose external radiation.

**Conclusion:**

Different factors come into account for polyorchidsm management: the drainage system, the fertile potential of the supernumerary gonad, and its localization. In cases of uncomplicated polyorchidism, a conservative treatment, with US or MRI follow-up seems to be a rational choice without surgical complications.

## Highlights

• Polyorchidism is a rare congenital abnormality. To the best of our knowledge, this is the first report of leiomyoma in supranumerary testis (SNT).
• Most cases of polyorchidism are found incidentally during surgery for inguinal hernia, undescended testes, torsion or testicular tumor.
• There is no consensus regarding the management of SNT. If the SNT is scrotal, most authors recommend conservative management.
• If nonscrotal SNT is found incidentally during surgery, orchiectomy could be performed because of increased risk of malignancy.
• Treatment of intratubular germ cell neoplasia includes surveillance, orchiectomy, or low-dose external radiation.

## 1 Introduction

Polyorchidism, defined as the presence of more than two testicles, is a rare congenital abnormality of the male genital tract. Triorchidism is the most common variant. Two important classifications of polyorchidism are the embryological one of Leung and the vascular one of Bergholz. Usually asymptomatic, it can be revealed by an abdominal mass, a testicular torsion or an inguinal hernia. Its management is controversial depending on different factors. To the best of our knowledge, we report the first case of leiomyoma in supranumerary testis. We summarize also the classifications and management of polyorchidism. Our work has been reported in line with the SCARE criteria [1].

## 2 Presentation of case

A 41-year-old man presented to our institute for bilateral inguinal swelling and infertility for 15 years. He stated that his scrotum was always empty since childhood. There was no other medical history.

Physical examination found a palpable right testis in the right lower abdomen along with an empty scrotum. The left testis was not palpable. Ultrasonography (US) showed both undescended right and left testes respectively in the right and left inguinal canals. Both testes had heterogeneous echogenicity. Right testis measured 45 × 30 × 13 mm while left testis measured 24 × 20 × 10 mm.

Due to malignancy risk we decided to do an orchiopexy with testicular biopsy one side at a time. Pre-operative serum tumor markers (Lactate dehydrogenase, beta human chorionic gonadotropin, alpha foetoprotein) were normal.

**Fig. 1:**
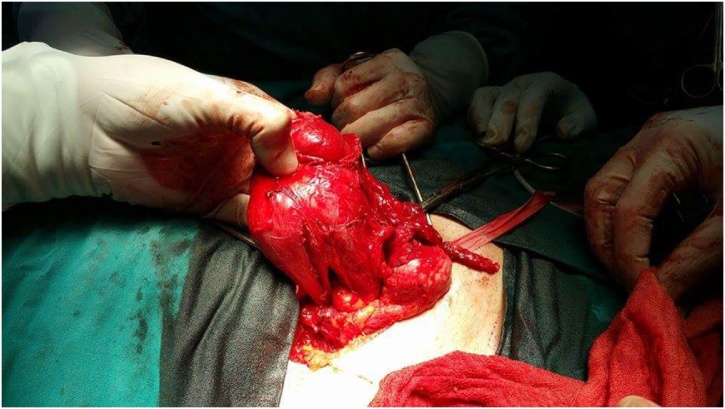
Intra-operative findings after right inguinal incision.

**Fig. 2:**
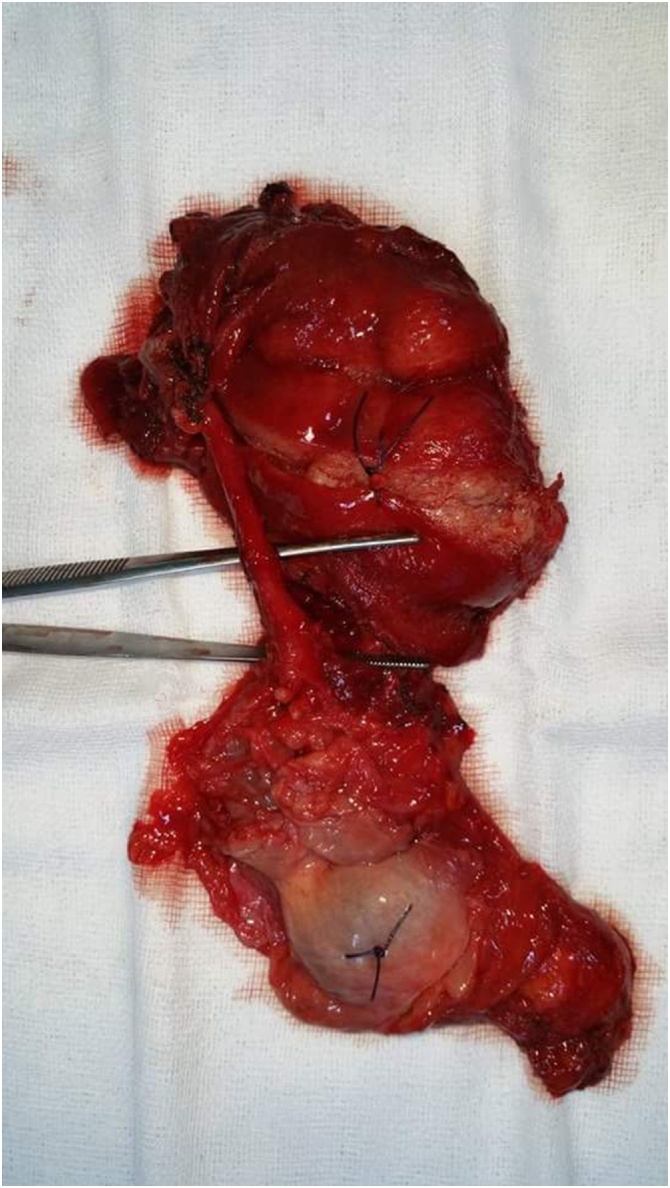
The two right testes after orchiectomy. The inferior testis in the image is dysmorphic and suspicious, while the superior testis seemes macroscopically normal. The inferior testis drains into the epididymis of the superior testis with a common vas deferens.

He was operated upon through right inguinal incision. There was no hernia sac. Two structures, identified as two right testes, were sharing a common vas deferens and a double separate epididymides. The superior testis measured 80 × 50 × 35 mm and was highly dysmorphic and suspicious, while the lower testis measured 40 × 30 × 25 mm and seemed macroscopically normal. The superior testis drains into the epididymis of the lower testis. Orchiectomy for the two right testes was performed (Fig. 1, Fig. 2). Pathology examination confirmed the testicular nature of the two structures and found that the dysmorphic superior testis was the site of a large leiomyoma and the lower tesits was the site of an intratubular germ cell neoplasia (IGCN).

**Fig. 3:**
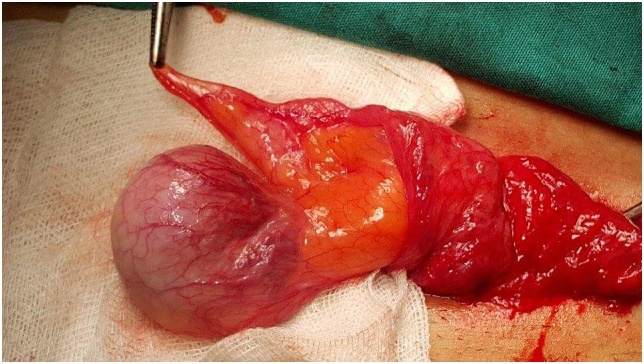
A complete separation between the epididymis and the left testis with a mesentery. If nonscrotal SNT is found incidentally during surgery, orchiectomy could be performed because of increased risk of malignancy

We planned for surgical exploration directed to the left-sided cryptorchidism by an inguinal approach. An eutrophic and macroscopically normal left testis was found. A complete separation between the epididymis and the testis with a mesentery between them was noted (Fig. 3). Testicular biopsies followed by a left orchiopexy in dartos were performed. Histopathological examination of the specimen was compatible with IGCN. We opted for surveillance with follow-up and regular testicular self-examination and US.

## 3 Discussion

Polyorchidism is rare with around 200 cases reported in the literature to date [2]. Leung described the first classification by embryological development with functional implications as follows:

-**type I:** supernumerary testis (SNT) without epididymis and vas deference.

**-type II:** SNT shares common epididymis and vas deference with ipsilateral testis.

**-type III:** SNT has its own epididymis but shares a common vas deferens with the ipsilateral testis.

**-type IV:** complete duplication of testis, epididymis and vas.

According to Leung, types II and III together represent more than 90% of the cases [3].

Bergholz and Wenke proposed an innovative classification. A testis being drained by an outflow path was coded as **type A**. Undrained testes without connection to a draining vas deferens were coded as **type B**. Type A testes were further divided into the subgroups A1 (own epididymis and vas deferens), A2 (own epididymis but common vas deferens), A3 (common epididymis and vas deferens), A4 (own vas deferens but common epididymis) and AX (no further description of appendixes). Type B testes were divided into the subgroups B1 (own epididymis), B2 (no epididymis, thus testicular tissue only) and BX (no further description of appendixes) [4]. Our case was compatible with Type III according to Leung and A2 according to Bergholz.

Most cases of polyorchidism are asymptomatic and are found incidentally during surgery for inguinal hernia (30%), undescended testes (15–30%) as in our case, torsion (13%), hydrocele (9%) or testicular tumor [2]. In a series of 140 polyorchidism cases, Bergholz [4] reported 10 cases of testicular tumor (7.14%): 2 were benign (rete testis adenoma) and 8 were malignant (3 seminomas, 2 choriocarcinomas, 2 teratomas, 1 embryonal carcinoma). 7 of these 8 cases were undescended SNT. To the best of our knowledge, this is the first report of leiomyoma in SNT.

There is no consensus regarding the management of SNT due to its rareness. If the SNT is scrotal, confirmed by ultrasound or magnetic resonance imaging (MRI), and there is no other indication for surgery, most authors recommend conservative management with regular ultrasound follow-up [2,4,5]. If nonscrotal SNT is found incidentally during surgery, orchiectomy could be performed because of increased risk of malignancy [2,4]. However, type A testes are best treated with orchiopexy to prevent future torsion and allow follow-up by US and self-examination. Biopsy of the SNT will prove the histology and provide an evaluation of the degree of spermatogenesis. For our patient, the two right testes were managed by orchiectmy. Histopathological examination corresponded to leiomyoma for the supernumerary testis and to intratubular germ cell neoplasia (IGCN) for the usual right and left testes. Testicular leiomyoma is rare and may be confused with malignancy. IGCN has been reported to be less than 1% in the normal male population, and 0.43% to 0.8% in autopsy studies, with most cases being unilateral [6].

Treatment of IGCN includes surveillance, orchiectomy, or low-dose external radiation (LDRT). For patients with unilateral IGCN and a normal contralateral testis, orchiectomy is the preferred treatment option. IGCN in solitary testis is more controversial, with some advocating surveillance with testicular self-examination and others recommending early LDRT [6].

For our patient, we opted for conservative management with regular US and follow-up to avoid risks of hypogonadism.

## 4 Conclusion

The diagnosis of polyorchidism is often occasional.

Different factors come into account when choosing their management: the drainage system, the fertile potential of the supernumerary gonad, and its localization. Surgery should only be considered appropriate if there is an associated pathology. Indeed, in cases of uncomplicated polyorchidism, a conservative treatment, with US or MRI follow-up seems to be a rational choice without surgical complications.

## Conflicts of interest

NO financial and personal relationships with other people or organisations that could inappropriately influence (bias) their work.

## Sources of funding

No source of funding.

## Ethical approval

Ibn El Jazzar Teaching Hospital ethic comitee, Kairouan, Tunisia.

## Consent

Written informed consent was obtained from the patient for publication of this case report and accompanying images.

## Author contribution

**Boussaffa H**; concept or design, data collection, data analysis or interpretation, writing the paper.

**Naouar S**; concept or design, data collection, data analysis or interpretation, writing the paper.

**Ati N**; data collection, data analysis or interpretation.

**Amri M**; data collection.

**Ben Khelifa B**; data collection.

**Salem B**; data collection, data analysis or interpretation, writing the paper.

**El Kamel R**; writing the paper.

## Registration of research studies

This is no research study.

## Guarantor

Boussaffa Hamza & Ati Nidhal.

## Provenance and peer review

Not commissioned, externally peer reviewed.
